# TGF-β and TNF-α Signaling Crosstalk in Human Coronary Artery Cells

**DOI:** 10.3390/ijms27093948

**Published:** 2026-04-29

**Authors:** Klaudia Bonowicz-Kozłowska, Dominika Jerka, Damian Twardak, Konrad Kleszczyński, Maciej Gagat

**Affiliations:** 1 Department of Histology and Embryology, Collegium Medicum in Bydgoszcz, Nicolaus Copernicus University in Torun, 85-092 Bydgoszcz, Poland; klaudia.bonowicz@cm.umk.pl (K.B.-K.); dominika.jerka@cm.umk.pl (D.J.); damian.twardak@cm.umk.pl (D.T.); 2 Department of Morphological and Physiological Sciences, Faculty of Medicine, Collegium Medicum, Mazovian Academy in Płock, 09-402 Płock, Poland; 3 Department of Dermatology, University of Münster, Von-Esmarch-Str. 58, 48149 Münster, Germany; konrad.kleszczynski@ukmuenster.de

**Keywords:** TGF-β1, TNF-α, coronary artery endothelial cells, coronary artery smooth muscle cells

## Abstract

Transforming growth factor-β1 (TGF-β1) and tumor necrosis factor-α (TNF-α) are central regulators of vascular inflammation and remodeling in coronary artery disease. However, their cell-type-specific and context-dependent effects in primary human coronary artery endothelial cells (ECs) and vascular smooth muscle cells (VSMCs) remain incompletely defined. Primary human coronary artery endothelial cells (pHCAECs) and smooth muscle cells (pHCASMCs) were stimulated with TGF-β1 (10 ng/mL), TNF-α (100 ng/mL), or their combination. Canonical SMAD2/3 activation, Krüppel-like factor 11 (KLF11) expression, cytoskeletal and junctional remodeling, vascular cell adhesion molecule-1 (VCAM-1) expression, migration dynamics (wound healing and confluent assays), and endothelial tube formation were assessed using immunofluorescence microscopy, live-cell imaging, and quantitative trajectory analysis. Both cytokines were associated with increased nuclear pSMAD2/3 signal in ECs and VSMCs, consistent with functional interplay between inflammatory and TGF-β-related signaling pathways. In pHCAECs, TNF-α robustly induced VCAM-1 functional expression and disrupted VE-cadherin continuity, whereas TGF-β1 primarily promoted cytoskeletal remodeling without strong inflammatory activation. TGF-β1 increased endothelial migration velocity and accumulated distance. In contrast, TNF-α preferentially enhanced Euclidean displacement and directional persistence, shifting the migratory pattern toward more directed movement most evident under combined TGF-β1 + TNF-α stimulation. Notably, TGF-β1 significantly reduced endothelial tube formation, indicating impaired network organization rather than proangiogenic activity. In pHCASMCs, TGF-β1 enhanced migratory activity, particularly in confluent monolayers, whereas TNF-α enhanced directional displacement. KLF11 was induced by TGF-β1 in both pHCAECs and pHCASMCs. In pHCAECs, TNF-α also increased KLF11 and co-stimulation promoted nuclear enrichment, whereas in pHCASMCs TNF-α alone was not effective and combined treatment amplified the TGF-β1 response, supporting cell-type-specific integration of inflammatory and TGF-β-dependent signals. TGF-β1 and TNF-α differentially regulate the inflammatory activation and migration of primary human coronary vascular cells in a cell-type- and structural-context-dependent manner. TGF-β1 enhances migratory force generation, whereas TNF-α reinforces directional polarization, and their integration determines effective vascular repair dynamics. Canonical SMAD2/3 activation does not uniformly predict functional outcome, and KLF11 was identified as a context-sensitive transcription-associated factor showing differential nuclear localization in response to cytokine stimulation, representing a hypothesis-generating observation for future mechanistic studies.

## 1. Introduction

Cardiovascular diseases remain the leading cause of mortality worldwide, with coronary artery atherosclerosis constituting their principal pathological basis. Although atherosclerosis was initially viewed primarily as a disorder of lipid metabolism, extensive experimental and clinical evidence has established that inflammatory processes are fundamentally involved in its pathogenesis. Accordingly, atherosclerosis is now recognized as a chronic inflammatory disease of the vascular wall. Within this framework, vascular-resident cells, particularly endothelial cells (ECs) and vascular smooth muscle cells (VSMCs), play a central role in regulating disease development. Under inflammatory conditions, these cells become activated, undergo phenotypic changes, and engage in intercellular signaling that contributes to lesion initiation, progression, and plaque destabilization [1]. These functional changes are accompanied by the dynamic reorganization of filamentous actin (F-actin), which is a key determinant of endothelial cell morphology and mechanical properties. In the resting endothelium, F-actin is predominantly organized into a cortical network that supports junctional stability, whereas inflammatory stimulation promotes its reorganization into contractile stress fibers. This cytoskeletal remodeling alters intracellular tension and contributes to changes in cell shape, barrier integrity, and endothelial activation [2,3]. Importantly, despite effective lipid-lowering therapy, inflammatory activity often persists, as indicated by elevated inflammatory biomarkers. This “residual inflammatory risk” is strongly associated with plaque instability and acute coronary syndromes, underscoring inflammation’s contribution to atherosclerotic disease progression independent of lipid burden [4].

Among the cytokines shaping the inflammatory microenvironment of the coronary artery wall, transforming growth factor beta (TGF-β) and tumor necrosis factor alpha (TNF-α) have emerged as key regulators of EC and VSMC function [5]. TNF-α is a prototypical pro-inflammatory cytokine that plays a central role in the initiation and progression of atherosclerosis [6]. In coronary artery endothelial cells, TNF-α activates inflammatory signaling pathways, leading to the increased expression of adhesion molecules and chemokines, the promotion of leukocyte recruitment, and the impairment of endothelial barrier integrity. In vascular smooth muscle cells, TNF-α promotes phenotypic modulation toward a more synthetic and inflammatory state, associated with increased migratory potential and reinforced inflammatory signaling within the vessel wall [7]. In contrast, the role of TGF-β in coronary atherosclerosis is highly context-dependent and remains incompletely understood. Although traditionally regarded as an anti-inflammatory and vasoprotective cytokine, TGF-β exerts pleiotropic and sometimes opposing effects within the vascular wall, depending on ligand concentration, duration of exposure, cellular target, and the inflammatory microenvironment. Through canonical SMAD-dependent signaling, TGF-β contributes to vascular homeostasis by regulating extracellular matrix turnover and limiting excessive immune activation; however, the dysregulated or sustained activation of canonical and non-canonical pathways has been linked to endothelial dysfunction, endothelial-to-mesenchymal transition, and pathological vascular remodeling. In coronary artery vascular smooth muscle cells, TGF-β regulates extracellular matrix synthesis, migration, and phenotypic plasticity, thereby influencing fibrous cap formation and plaque stability, while excessive or aberrant signaling can drive fibrosis and maladaptive remodeling. Despite extensive experimental and clinical investigation, critical uncertainties remain regarding the cell-specific and dose-dependent effects of TGF-β in coronary atherosclerosis, as many mechanistic insights derive from animal models or immortalized cell lines that may not fully recapitulate human coronary vascular biology, underscoring the need for physiologically relevant studies using primary human cells [8,9]. Importantly, the cellular effects of both TNF-α and TGF-β are mediated, at least in part, through the regulation of transcriptional programs that integrate inflammatory and remodeling signals. Several transcription factors have been identified as shared downstream targets of these cytokines, enabling the coordinated control of the vascular cell phenotype. Among them, Krüppel-like factor 11 (KLF11) has emerged as a TGF-β-responsive transcription factor that modulates inflammatory gene expression and cellular differentiation [10]. Evidence indicates that KLF11 expression is induced by TGF-β signaling and can interact functionally with inflammatory pathways, including those activated by TNF-α, supporting the possibility that KLF11 may represent a molecular link between cytokine-driven inflammation and transcriptional regulation in vascular cells. Such convergence at the level of transcriptional control may contribute to EC and VSMC responses during chronic vascular inflammation [11].

Emerging evidence indicates that TGF-β and TNF-α signaling may coexist and interact within chronically inflamed coronary vessels. However, the extent to which these cytokines differentially regulate inflammatory signaling and functional responses in coronary artery endothelial cells compared with vascular smooth muscle cells remains poorly defined. This study aimed to investigate the effects of TGF-β and TNF-α on the inflammatory activation of coronary artery endothelial cells and coronary artery smooth muscle cells, with particular emphasis on differences in the signaling pathways involved and the profile of the inflammatory response. A better understanding of the cell-specific mechanisms regulating inflammation in the coronary vessel wall may provide a new basis for designing targeted therapeutic strategies aimed at modulating vascular inflammation in coronary artery disease.

## 2. Results

### 2.1. Assessment of Cell Viability Following Stimulation with TNF-α and Increasing Concentrations of TGF-β1

**Figure 1:**
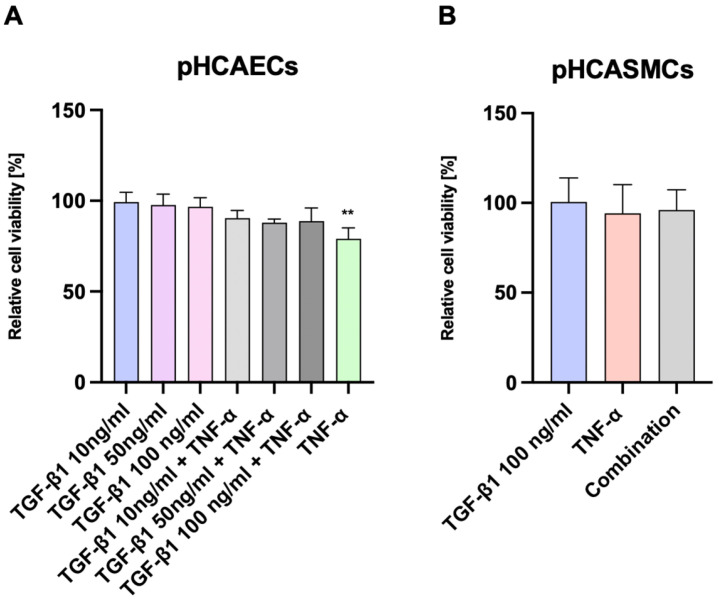
The effect of TNF-α and TGF-β1 on coronary artery cell viability. (**A**) The relative viability of the primary human coronary artery endothelial cells (pHCAECs) after 24 h stimulation with recombinant human TGF-β1 (10, 50, or 100 ng/mL) alone or in combination with tumor necrosis factor-α (TNF-α) (100 ng/mL), assessed using the 3-(4,5-dimethylthiazol-2-yl)-2,5-diphenyltetrazolium bromide (MTT) assay. Treatment with TNF-α alone resulted in a significant reduction in endothelial cell viability compared to control conditions. (**B**) The relative viability of the primary human coronary artery smooth muscle cells (pHCASMCs) after stimulation with TGF-β1 (100 ng/mL), TNF-α (100 ng/mL), or their combination. No significant changes in pHCASMC viability were observed under any of the conditions studied. The data are presented as mean ± SD. Statistical analysis was performed using one-way analysis of variance (ANOVA) and Dunnett’s multiple comparison test. The cell viability values were normalized to the untreated control cells, which were set to 100% viability and served as the reference baseline for all the comparisons. ** *p* < 0.01 compared to the control group.

Prior to functional and signaling analyses, an MTT assay was performed to evaluate the impact of TNF-α and TGF-β1 on the viability of primary human coronary artery endothelial cells (pHCAECs) and coronary artery smooth muscle cells (pHCASMCs), in order to exclude the potential cytotoxic effects of the applied cytokine treatments. Cell viability values were normalized to the untreated control cells included in each experiment, which were defined as 100% viability and served as the reference condition for all statistical comparisons (Figure 1). The purpose of this experiment was twofold: first, to confirm that the inflammatory dose of TNF-α used throughout the study does not induce excessive cytotoxicity; and second, to determine whether exposure to high concentrations of TGF-β1 applied deliberately to model cytokine excess affects cell viability, either alone or under inflammatory conditions. TNF-α was applied at a constant concentration of 100 ng/mL, which had been previously established experimentally as an effective dose inducing inflammatory activation in endothelial cells [5]. The selected range of TGF-β1 concentrations (10–100 ng/mL) was based on published studies demonstrating the effective activation of TGF-β signaling in vascular cells and was intentionally chosen to model the conditions of sustained inflammatory stimulation rather than minimal physiological activation. pHCASMCs were included as a mechanistic control cell type and were exposed to cytokine concentrations selected on the basis of endothelial cell responses, allowing the comparison of cell-type-specific effects under identical experimental conditions. In the pHCAECs, treatment with TGF-β1 alone at concentrations of 10, 50, or 100 ng/mL for 24 h did not significantly alter cell viability compared with control cells, as determined by Dunnett’s multiple comparisons test (Figure 1A). Similarly, co-treatment with TNF-α and increasing concentrations of TGF-β1 did not result in a statistically significant reduction in endothelial cell viability relative to control conditions. In contrast, stimulation with TNF-α alone caused a significant decrease in pHCAEC viability compared with the control cells (adjusted *p* = 0.0024), confirming the biological activity of TNF-α while indicating that the observed effect was not further exacerbated by additional TGF-β1 exposure. In the pHCASMCs, no significant differences in cell viability were observed following treatment with TGF-β1 (10 ng/mL), TNF-α, or their combination (Figure 1B). All experimental conditions resulted in metabolic activity comparable to that of control cells, indicating that coronary artery smooth muscle cells are relatively resistant to cytokine-induced viability changes under the tested conditions. Collectively, these results demonstrate that exposure to high concentrations of TGF-β1 does not compromise the viability of either endothelial or smooth muscle cells, even in the presence of inflammatory stimulation. Importantly, this confirms that subsequent analyses of inflammatory signaling and functional responses reflect genuine cytokine-mediated effects rather than secondary consequences of reduced cell viability.

### 2.2. Activation of the TGF-β/SMAD2/3 Pathway and Cytoskeletal Reorganization in pHCAECs

**Figure 2:**
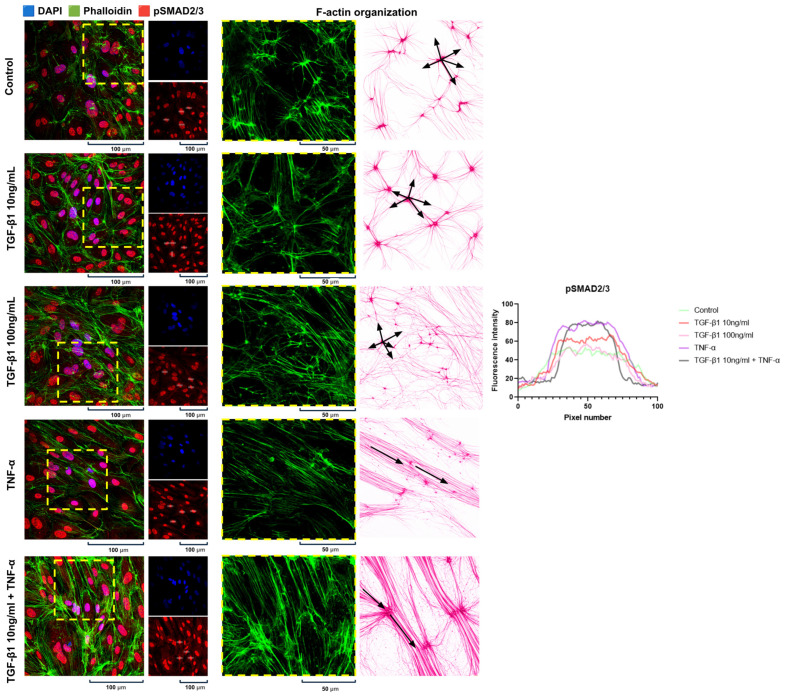
The activation of the canonical transforming growth factor-β (TGF-β)/SMAD family member 2/3 (SMAD2/3) signaling pathway and cytoskeletal remodeling in the pHCAECs following cytokine stimulation (magnification ×1000). Representative immunofluorescence images of the pHCAECs treated with TGF-β1 (10 ng/mL or 100 ng/mL), (TNF-α), or their combination for 24 h. The nuclei were stained with 4′,6-diamidino-2-phenylindole (DAPI, blue) and filamentous actin (F-actin) with phalloidin (green), and phosphorylated SMAD2/3 (pSMAD2/3) is shown in red. The nuclear accumulation of pSMAD2/3 was observed under all the stimulatory conditions, with the highest fluorescence intensity detected following TNF-α treatment and combined TGF-β1 + TNF-α stimulation. TGF-β1 (10 ng/mL) increased the nuclear pSMAD2/3 intensity compared with control, whereas a higher TGF-β1 concentration (100 ng/mL) was associated with a reduced fluorescence signal. The phalloidin staining demonstrated pronounced actin cytoskeletal remodeling in response to TNF-α and combination treatment, characterized by enhanced stress fiber formation. The fluorescence intensity was quantified using ImageJ software by measuring the pixel intensity values within defined regions of interest (ROIs); the representative ROIs used for analysis are indicated by the white squares. The fluorescence intensity profile (right panel) illustrates pixel distribution across selected regions. The black arrows indicate representative actin structures and their organization, reflecting the distribution of intracellular tensions.

The selection of cytokine concentrations was guided by an evaluation of TGF-β/SMAD2/3 pathway activation; to assess canonical signaling, the nuclear localization and fluorescence intensity of phosphorylated SMAD2/3 (pSMAD2/3) were quantified using pixel-based analysis within defined nuclear regions of interest, demonstrating low basal fluorescence in control cells, moderate nuclear accumulation following TGF-β1 stimulation at 10 ng/mL, the attenuation of nuclear signal at higher TGF-β1 concentrations, and the highest peak fluorescence intensity in response to TNF-α treatment, which reached statistical significance compared with control in a one-way ANOVA followed by Dunnett’s test (adjusted *p* = 0.0002), while the combined TGF-β1 + TNF-α condition exhibited elevated nuclear pSMAD2/3 levels characterized by a broad high-intensity plateau on the fluorescence intensity profile but did not reach statistical significance, likely reflecting variability in nuclear size and signal distribution rather than absence of pathway activation (Figure 2). These data indicate increased nuclear pSMAD2/3 accumulation under the selected experimental conditions, consistent with engagement of canonical TGF-β-related signaling, and support the use of these cytokine concentrations for subsequent functional analyses.

### 2.3. Morphological Assessment of pHCAECs in a Confluent Monolayer

**Figure 3:**
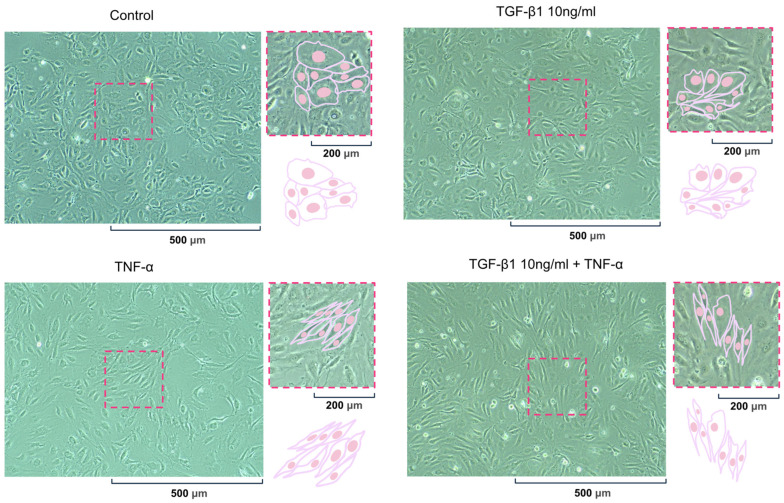
The morphology of the pHCAECs in a confluent monolayer following 24 h cytokine stimulation (magnification ×200. The bright-field phase-contrast micrographs of the pHCAECs cultured to near 100% confluence and treated for 24 h with TGF-β1 (10 ng/mL), TNF-α (100 ng/mL), or their combination. The control cells exhibited predominantly polygonal morphology typical of confluent endothelial monolayers. TGF-β1 treatment induced mild cellular elongation, whereas TNF-α stimulation resulted in a more pronounced spindle-like phenotype. The most evident elongation and increased cellular alignment were observed following the combined TGF-β1 and TNF-α treatment. Monolayer continuity was preserved under all conditions. Magnification ×200. The schematic insets illustrate representative cell morphology and the alignment patterns characteristic of each condition.

A morphological evaluation of the pHCAECs cultured under near-confluent conditions (100% confluence) for 24 h following stimulation with TGF-β1 (10 ng/mL), TNF-α (100 ng/mL), or their combination was performed using bright-field phase-contrast microscopy. Under control conditions, the pHCAECs formed a compact monolayer with predominantly polygonal morphology and preserved intercellular contacts. The cells displayed a typical endothelial appearance at high confluence. TGF-β1 stimulation induced mild morphological changes, including the moderate elongation of selected cells, while overall monolayer organization remained preserved. In contrast, TNF-α-treated pHCAECs exhibited marked cellular elongation and a more spindle-like phenotype. The classical cobblestone morphology was reduced, and cells appeared stretched along their longitudinal axis. The most pronounced morphological alterations were observed following combined TGF-β1 and TNF-α treatment. The cells demonstrated evident elongation, increased alignment, and a more anisotropic organization compared with control and single-cytokine conditions. Despite these changes, monolayer continuity was maintained, and no significant cell detachment was observed. Collectively, TNF-α alone and in combination with TGF-β1 induced a clear shift toward an elongated endothelial phenotype under confluent conditions (Figure 3).

### 2.4. Cytokine-Induced Changes in the Expression and Localization of Functionally Relevant Proteins in pHCAECs

**Figure 4:**
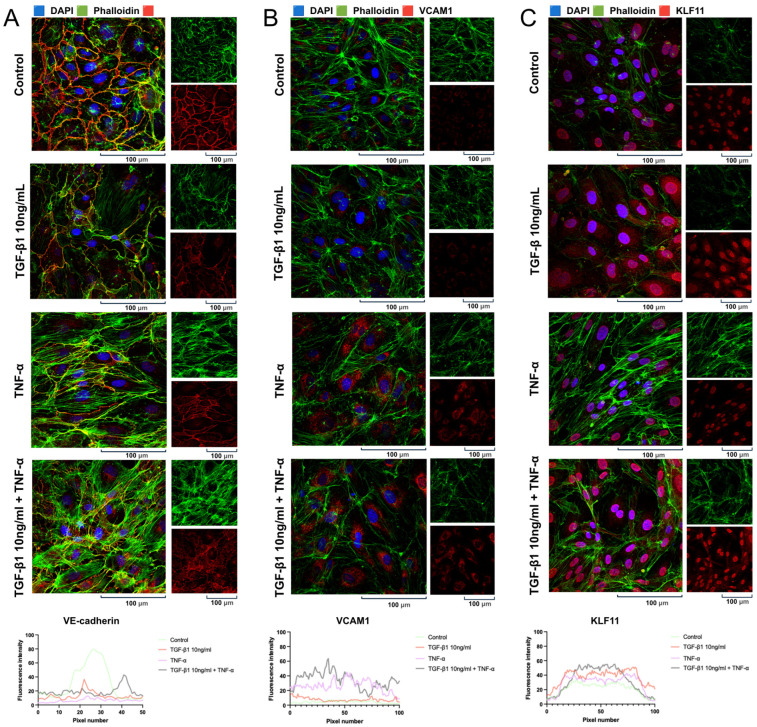
Cytokine-induced cytoskeletal remodeling, junctional reorganization, and protein localization and fluorescence signal changes in pHCAECs (magnification ×1000). Representative immunofluorescence images of pHCAECs treated for 24 h with TGF-β1 (10 ng/mL), TNF-α (100 ng/mL), or their combination; nuclei were stained with DAPI (blue), F-actin with phalloidin (green), and vascular endothelial cadherin (VE-cadherin) (**A**), vascular cell adhesion molecule-1 (VCAM-1) (**B**), or Krüppel-like factor 11 (KLF11) (**C**) are shown in red; cytokine stimulation induces disruption of VE-cadherin junctional continuity, upregulation of VCAM-1, and increased nuclear localization of KLF11, accompanied by a shift from cortical actin organization to prominent stress fiber formation; insets show magnified regions and bottom panels present fluorescence intensity profiles; images are representative of independent experiments.

An immunofluorescence analysis of the pHCAECs revealed marked cytokine-dependent alterations in the cytoskeletal organization and intercellular junction integrity. In control conditions, the cells displayed a typical endothelial morphology characterized by a well-organized cortical F-actin network and continuous VE-cadherin localization at the cell–cell borders, forming a characteristic cobblestone-like monolayer. Treatment with TGF-β1 (10 ng/mL) induced visible cytoskeletal remodeling, manifested by the increased formation of actin stress fibers and the partial redistribution of F-actin from the cortical region toward bundled intracellular filaments. Concomitantly, VE-cadherin staining appeared less uniformly distributed along the intercellular junctions, suggesting early junctional reorganization. TNF-α stimulation resulted in pronounced actin polymerization and prominent stress fiber formation aligned along the longitudinal axis of the elongated cells. Under these conditions, VE-cadherin continuity at the cell borders was visibly disrupted, with reduced membrane localization and a more fragmented staining pattern, indicating compromised endothelial junction integrity. The combined treatment with TGF-β1 (10 ng/mL) and TNF-α produced the most prominent structural alterations. The cells exhibited extensive stress fiber formation and enhanced actin bundling, accompanied by a marked reduction in continuous VE-cadherin localization at intercellular contacts. The merged images demonstrated a clear shift from cortical actin organization toward a contractile phenotype associated with cytoskeletal tension and junctional destabilization (Figure 4A). A quantitative fluorescence intensity analysis confirmed these observations, showing a significant increase in signal intensity in all stimulated conditions compared with control (Dunnett’s multiple comparisons test: CTRL vs. TGF-β1, mean difference = 18.01, 95% CI 11.78–24.24, *p* < 0.0001; CTRL vs. TNF-α, mean difference = 24.44, 95% CI 18.21–30.67, *p* < 0.0001; CTRL vs. combined treatment, mean difference = 11.78, 95% CI 5.552–18.01, *p* < 0.0001). Collectively, these findings indicate that inflammatory and profibrotic stimulation induces significant cytoskeletal remodeling and the disruption of adherens junction organization in pHCAECs, consistent with endothelial activation and loss of barrier integrity.

Immunofluorescence analysis was performed to assess VCAM-1 expression in the pHCAECs (Figure 4B) following 24 h stimulation with TGF-β1 (10 ng/mL), TNF-α, or their combination. Under control conditions, VCAM-1 expression was low and limited to weak intracellular staining. Treatment with TGF-β1 alone did not result in a pronounced increase in VCAM-1 signal compared with the control cells. In contrast, TNF-α stimulation markedly upregulated VCAM-1 expression, as evidenced by a substantial increase in fluorescence intensity and enhanced intracellular distribution. A similarly elevated VCAM-1 signal was observed following combined TGF-β1 and TNF-α treatment, indicating sustained endothelial activation under inflammatory conditions. Quantitative analysis confirmed that TGF-β1 alone did not significantly alter VCAM-1 expression compared with control (Dunnett’s test: mean difference = −1.12, 95% CI −3.85 to 1.61, *p* = 0.42), whereas TNF-α treatment resulted in a highly significant increase in VCAM-1 fluorescence intensity (mean difference = −22.24, 95% CI −24.57 to −19.90, *p* < 0.0001). Similarly, combined TGF-β1 and TNF-α stimulation produced a strong and significant upregulation of VCAM-1 expression (mean difference = −30.10, 95% CI −32.43 to −27.76, *p* < 0.0001). These observations demonstrate that TNF-α is the principal driver of VCAM-1 induction in pHCAECs, while TGF-β1 alone does not strongly stimulate VCAM-1 expression (Figure 4). The combined treatment maintains high VCAM-1 levels, consistent with a pro-inflammatory endothelial phenotype.

Immunofluorescence analysis demonstrated that KLF11 exhibited predominantly nuclear localization in the pHCAECs under all experimental conditions. Quantitative fluorescence profiling revealed a significant increase in KLF11 signal intensity following TGF-β1 stimulation (10 ng/mL) compared with the control cells (mean difference = −16.07; 95% CI −20.23 to −11.91; adjusted *p* < 0.0001). TNF-α treatment also significantly increased KLF11 fluorescence intensity relative to control (mean difference = −7.400; 95% CI −11.56 to −3.244; adjusted *p* < 0.0001), although the magnitude of induction was lower than that observed with TGF-β1 alone. Notably, combined TGF-β1 + TNF-α stimulation resulted in a robust upregulation of KLF11 compared with control (mean difference = −11.87; 95% CI −16.03 to −7.715; adjusted *p* < 0.0001). Importantly, a qualitative analysis of the immunofluorescence images revealed a pronounced increase in nuclear KLF11 signal intensity under cytokine stimulation, which was particularly evident in the combined treatment group. In these cells, a strong nuclear accumulation of KLF11 was observed in a substantial proportion of nuclei, indicating enhanced transcriptionally active localization. Collectively, these findings indicate that both TGF-β1 and TNF-α are associated with increased KLF11 signal intensity in endothelial cells, with combined stimulation producing the most pronounced nuclear enrichment (Figure 4C).

### 2.5. Effects of Cytokine Stimulation on Migration of pHCAECs in the Wound Healing Assay

**Figure 5:**
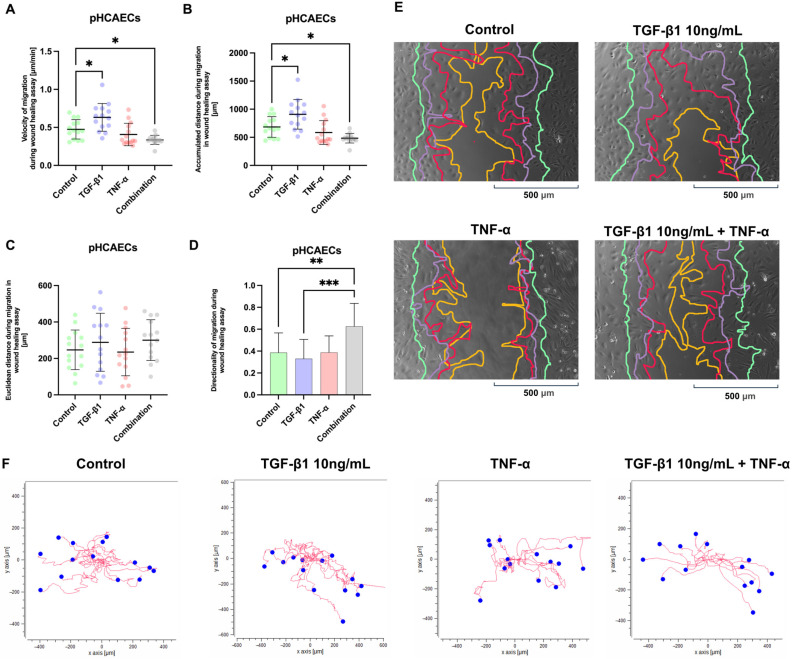
Cytokine-dependent modulation of pHCAEC migration in the wound healing assay. (**A**) Migration velocity and (**B**) accumulated migration distance of pHCAECs following stimulation with TGF-β1 (10 ng/mL), TNF-α (100 ng/mL), or their combination. TGF-β1 significantly increased both migration velocity and accumulated distance compared with control cells, whereas TNF-α attenuated this promigratory effect. Combined treatment resulted in intermediate values. (**C**) Euclidean distance, reflecting net cell displacement from the origin, did not differ significantly among groups. (**D**) Directionality analysis demonstrated increased directional persistence in the combined treatment group compared with selected single-cytokine conditions. Data are presented as individual cell tracks with mean ± SD. Statistical analysis was performed using ordinary one-way ANOVA followed by Tukey’s multiple comparisons test. * *p* < 0.05, ** *p* < 0.01, *** *p* < 0.001. (**E**) Representative images of endothelial wound closure under control conditions and after stimulation with TGF-β1 (10 ng/mL), TNF-α (100 ng/mL), or their combination. The contours of the wound area are marked at successive time points: 3 h (green), 6 h (purple), 12 h (red), and 24 h (yellow). The progressive displacement of the wound edge over time reflects endothelial cell migration. (**F**) Lower panels show individual cell tracking plots; red lines indicate migration paths, and blue dots mark starting positions. Axes are presented in micrometers (µm).

The migration of pHCAECs was assessed using a wound healing assay, and four parameters were quantified: migration velocity (Figure 5A), accumulated migration distance (Figure 5B), Euclidean distance (Figure 5C), and directionality (Figure 5D). An analysis of the migration velocity (Figure 5A) demonstrated a significant overall difference among the experimental groups (ordinary one-way ANOVA: F = 8.507, *p* < 0.0001; R^2^ = 0.3529). TGF-β1 stimulation significantly increased the migration velocity of the pHCAECs compared with the control cells (Tukey’s test: mean diff. = 226.0; 95% CI: 13.35 to 438.7; adjusted *p* = 0.0306). In contrast, TNF-α treatment resulted in a lower migration velocity relative to TGF-β1-treated cells (mean diff. = 323.6; 95% CI: 110.9 to 536.3; adjusted *p* = 0.0004) and values comparable to control conditions (adjusted *p* = 0.7620). Combined stimulation attenuated the promigratory effect observed with TGF-β1 alone (TGF-β1 vs. combination: mean diff. = 425.5; 95% CI: 212.8 to 638.2; adjusted *p* < 0.0001). Consistent with these findings, accumulated migration distance (Figure 5B) was significantly greater in the TGF-β1-treated pHCAECs than in the control or TNF-α-treated cells, with a significant difference between the TGF-β1 and combination groups (mean diff. = 0.2959; 95% CI: 0.08036 to 0.5114; adjusted *p* = 0.0019), and between the TNF-α and combination groups (mean diff. = 0.2381; 95% CI: 0.02255 to 0.4536; adjusted *p* = 0.0217); control vs. combination also reached significance (mean diff. = 0.2400; 95% CI: 0.02444 to 0.4555; adjusted *p* = 0.0202). TNF-α reduced accumulated migration distance relative to TGF-β1 stimulation, whereas combined treatment resulted in intermediate values. In contrast, Euclidean distance (Figure 5C), reflecting net displacement from the point of origin, did not differ significantly among groups (all adjusted *p* > 0.05), indicating that overall directional displacement was comparable despite differences in total migratory activity. The analysis of directionality (Figure 5D) revealed significant differences between the experimental conditions, with combined cytokine treatment showing greater directionality than control (mean diff. = 0.2180; 95% CI: 0.002508 to 0.4335; adjusted *p* = 0.0458) and TGF-β1 alone (mean diff. = 0.2959; 95% CI: 0.08036 to 0.5114; adjusted *p* = 0.0019). Figure 5E shows the wound healing images and single-cell trajectory plots (Figure 5F) of the pHCAECs under control conditions and after stimulation with TGF-β1, TNF-α, or their combination. Notably, combined TGF-β1 + TNF-α stimulation resulted in the most complete and cohesive wound closure, characterized by tightly advancing wound edges and increased directional persistence compared with control and single-cytokine conditions.

### 2.6. Migration of pHCAECs Under Confluent Conditions

**Figure 6:**
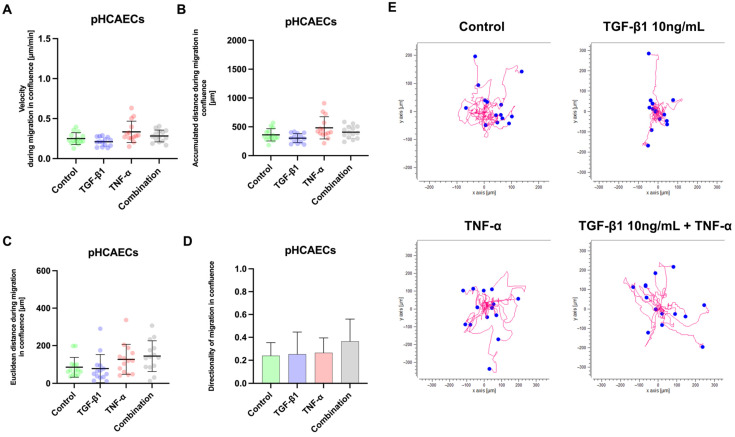
Migration of pHCAECs under confluent conditions following cytokine stimulation. (**A**) Migration velocity and (**B**) accumulated migration distance of pHCAECs cultured as a confluent monolayer and stimulated with TGF-β1 (10 ng/mL), TNF-α (100 ng/mL), or their combination. TNF-α treatment resulted in significantly higher migration velocity and accumulated distance compared with TGF-β1-treated cells, whereas other pairwise comparisons were not statistically significant. (**C**) Euclidean distance, reflecting net displacement from the point of origin, did not differ significantly among groups. (**D**) Directionality of migration also remained unchanged across experimental conditions. Data are presented as individual cell values with mean ± SD. Statistical analysis was performed using ordinary one-way ANOVA followed by Tukey’s multiple comparisons test (**E**) Representative migration trajectories of pHCAECs cultured as a confluent monolayer and treated with TGF-β1 (10 ng/mL), TNF-α (100 ng/mL), or their combination. Red lines represent individual cell migration paths over the observation period, and blue dots indicate starting positions. Axes are shown in micrometers (µm). TNF-α-treated cells display increased migratory dispersion compared with control and TGF-β1-treated cells, whereas combined stimulation results in intermediate migratory behavior. Trajectories are representative of independent experiments.

To assess endothelial cell motility under confluent conditions, migration velocity (Figure 6A), accumulated migration distance (Figure 6B), Euclidean distance (Figure 6C), and directionality (Figure 6D) were quantified in the pHCAECs stimulated with TGF-β1, TNF-α, or their combination. An analysis of migration velocity (Figure 6A) revealed a significant difference between the TGF-β1- and TNF-α-treated cells (Tukey’s test: mean diff. = −178.5; 95% CI: −307.6 to −49.41; adjusted *p* = 0.0031), with TNF-α inducing the highest migration velocity among the experimental groups, whereas the TGF-β1-treated cells exhibited reduced migratory speed. No significant differences were observed between control and TGF-β1 (*p* = 0.6454), control and TNF-α (*p* = 0.0725), control and combination (*p* = 0.7649), TGF-β1 and combination (*p* = 0.1516), or TNF-α and combination (*p* = 0.4322). Consistent with these findings, the accumulated migration distance (Figure 6B) was also significantly greater in the TNF-α-treated pHCAECs compared with the TGF-β1-treated cells (mean diff. = −0.1240; 95% CI: −0.2136 to −0.03431; adjusted *p* = 0.0031), whereas all remaining pairwise comparisons were not significant (control vs. TGF-β1, *p* = 0.6454; control vs. TNF-α, *p* = 0.0725; control vs. combination, *p* = 0.7649; TGF-β1 vs. combination, *p* = 0.1516; and TNF-α vs. combination, *p* = 0.4322). In contrast, Euclidean distance (Figure 6C) did not differ significantly among the groups (all comparisons ns), indicating comparable net displacement despite differences in overall migratory activity. Similarly, migration directionality (Figure 6D) remained statistically unchanged across experimental conditions (all comparisons ns), with relatively low directionality values consistent with collective migration in a confluent monolayer. Collectively, these results indicate that under confluent conditions TNF-α enhances the migratory activity of pHCAECs, as reflected by increased velocity and accumulated distance, whereas TGF-β1 does not promote endothelial migration in this context, and directional persistence remains largely unaffected by cytokine stimulation. Figure 6 demonstrates that TNF-α stimulation promotes greater spatial dispersion of pHCAEC migration under confluent conditions, while TGF-β1 restricts movement.

### 2.7. Cytokine-Induced Morphological Remodeling of pHCASMCs

**Figure 7:**
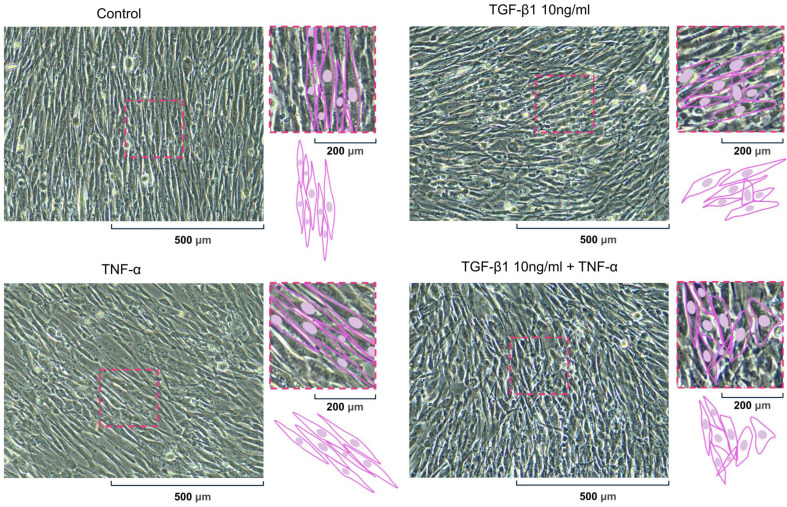
Cytokine-induced morphological changes in pHCASMCs. Representative phase-contrast micrographs of primary human coronary artery smooth muscle cells (pHCASMCs) following 24 h stimulation with TGF-β1 (10 ng/mL), TNF-α (100 ng/mL), or their combination. Control cells exhibited a typical elongated, spindle-shaped morphology with a highly parallel arrangement characteristic of the contractile phenotype of vascular smooth muscle cells. TGF-β1 stimulation was associated with increased cell density and slightly reduced uniformity of alignment while maintaining the spindle-shaped morphology. TNF-α treatment resulted in minor morphological changes, with cells remaining largely parallelly aligned and preserving overall monolayer organization. Combined stimulation with TGF-β1 and TNF-α led to increased cell overlap, denser packing, and greater heterogeneity in cell orientation, indicating context-dependent structural remodeling. Magnification ×200. Schematic insets illustrate representative cell morphology and alignment patterns characteristic of each condition.

A morphological assessment of the pHCASMCs after 24 h stimulation with TGF-β1 (10 ng/mL), TNF-α (100 ng/mL), or their combination was performed using phase-contrast microscopy. Under control conditions, the pHCASMCs exhibited the typical elongated, spindle-shaped morphology with a highly parallel arrangement and an organized growth pattern characteristic of vascular smooth muscle cells with a contractile phenotype. The cells formed a dense and uniformly oriented monolayer with a distinct longitudinal organization. Stimulation with TGF-β1 (10 ng/mL) induced subtle morphological changes. The pHCASMCs remained elongated; however, an increase in cell density and a slight reduction in the uniformity of alignment were observed. The cells appeared somewhat thicker and more closely packed, suggesting early phenotypic modulation while maintaining their overall spindle-shaped morphology. Treatment with TNF-α (100 ng/mL) resulted in minor morphological alterations. The cells remained elongated and largely parallelly aligned, with only slight variability in cell orientation and packing density compared with control conditions. No evident disruption of the overall monolayer organization was observed. Combined stimulation with TGF-β1 and TNF-α resulted in the most pronounced structural remodeling. The pHCASMCs displayed increased cell overlap and denser packing, accompanied by partial disruption of the previously uniform parallel architecture. The cells appeared more heterogeneous in shape and orientation compared with control conditions. Importantly, no significant cell detachment or overt cytotoxic effects were observed under any experimental condition (Figure 7).

### 2.8. Cytokine-Induced Changes in the Expression and Localization of Functionally Relevant Proteins in pHCASMCs

**Figure 8:**
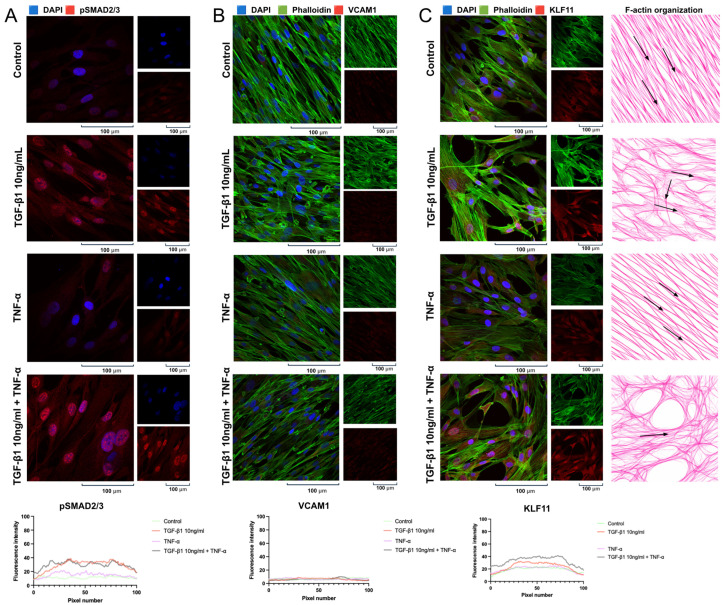
Cytokine-dependent activation of SMAD2/3 signaling and differential regulation of KLF11 and VCAM-1 expression in pHCASMCs (magnification ×1000). Representative immunofluorescence images of primary human coronary artery smooth muscle cells (pHCASMCs) after 24 h stimulation with TGF-β1 (10 ng/mL), TNF-α (100 ng/mL), or their combination, stained for pSMAD2/3 (red, panel (**A**)), VCAM-1 (red, panel (**B**)), KLF11 (red, panel (**C**)), F-actin (phalloidin, green), and nuclei (DAPI, blue); TGF-β1 induced strong nuclear localization of pSMAD2/3, TNF-α caused a modest increase, and combined treatment resulted in comparable activation; KLF11 expression was significantly increased following TGF-β1 and combined stimulation, while TNF-α alone had no significant effect; in contrast, VCAM-1 expression remained low under all conditions, with only a slight increase after TNF-α and no additional enhancement upon combined treatment; phalloidin staining additionally revealed cytokine-dependent cytoskeletal reorganization, reflecting changes in cell morphology, with quantitative analyses shown below each panel. Black arrows indicate representative actin structures and their organization, reflecting the distribution of intracellular tensions. Fluorescence intensity was interpreted as a relative readout of nuclear localization and pathway engagement.

Immunofluorescence analysis was performed to assess activation of the canonical TGF-β/SMAD2/3 pathway in pHCASMCs following 24 h stimulation with TGF-β1 (10 ng/mL), TNF-α (100 ng/mL), or their combination. In the control cells, pSMAD2/3 immunoreactivity was weak and predominantly cytoplasmic, with limited nuclear localization, indicating low basal pathway activity. Stimulation with TGF-β1 resulted in a marked increase in pSMAD2/3 signal intensity accompanied by clear nuclear accumulation. Quantitative analysis confirmed a significant elevation in fluorescence intensity compared with the control cells (mean difference [control − TGF-β1] = −17.58; 95% CI: −19.16 to −16.01; Dunnett’s multiple comparisons test, adjusted *p* < 0.0001; n = 100 per group). Treatment with TNF-α also significantly increased pSMAD2/3 fluorescence relative to control; however, the magnitude of this effect was substantially lower than that observed following TGF-β1 stimulation (mean difference [control − TNF-α] = −3.755; 95% CI: −5.333 to −2.177; adjusted *p* < 0.0001; n = 100). These findings indicate that inflammatory stimulation alone is associated with increased nuclear pSMAD2/3 signal, although to a markedly lesser extent than TGF-β1 stimulation. Combined treatment with TGF-β1 and TNF-α produced the highest pSMAD2/3 fluorescence intensity (mean difference [control − TGF-β1 + TNF-α] = −18.30; 95% CI: −19.88 to −16.72; adjusted *p* < 0.0001; n = 100), with pronounced nuclear accumulation visible in a substantial proportion of cells. The magnitude of activation was comparable to that induced by TGF-β1 alone (Figure 8A). Together, these findings demonstrate robust activation of canonical TGF-β/SMAD2/3 signaling in pHCASMCs upon cytokine stimulation, with TGF-β1 and combination inducing the strongest effect and TNF-α producing a modest increase.

The immunofluorescence analysis revealed overall low basal VCAM-1 expression in the pHCASMCs under control conditions. In contrast to the endothelial cells, the VCAM-1 signal in the smooth muscle cells was weak and predominantly diffuse, without prominent membrane localization. Quantitative analysis confirmed that stimulation with TGF-β1 (10 ng/mL) resulted in only a modest increase in VCAM-1 fluorescence intensity compared with the control cells; however, this effect did not reach statistical significance (mean difference: 0.5988; 95% CI: 0.2063 to 0.9912; adjusted *p* = 0.0011). Similarly, TNF-α treatment was associated with a slight enhancement of VCAM-1 signal, but this change was not statistically significant compared to control conditions (mean difference: 0.3352; 95% CI: −0.05721 to 0.7276; adjusted *p* = 0.1127). Combined TGF-β1 and TNF-α stimulation produced the highest increase in fluorescence intensity; however, when interpreted in the context of overall variability and biological relevance, this response remained modest and did not translate into a strong functional upregulation of VCAM-1 in the pHCASMCs (mean difference: 0.9190; 95% CI: 0.5266 to 1.311; adjusted *p* < 0.0001). Taken together, despite detectable trends toward increased VCAM-1 expression, all tested conditions showed only limited responsiveness relative to control, and the overall VCAM-1 signal in the pHCASMCs remained low. These findings indicate that coronary artery smooth muscle cells exhibit a substantially weaker and biologically less pronounced VCAM-1 response to inflammatory stimulation compared with endothelial cells under comparable experimental conditions (Figure 8B).

Immunofluorescence analysis was performed to evaluate KLF11 expression in the pHCASMCs following 24 h stimulation with TGF-β1 (10 ng/mL), TNF-α (100 ng/mL), or their combination. Under control conditions, KLF11 exhibited moderate basal expression with both cytoplasmic and nuclear localization (mean fluorescence intensity: 18.97). Stimulation with TGF-β1 significantly increased KLF11 fluorescence intensity compared with the control cells (mean: 23.74 vs. 18.97; mean difference [control − TGF-β1] = −4.772; 95% CI: −6.623 to −2.922; Dunnett’s multiple comparisons test, adjusted *p* < 0.0001; n = 100 per group), accompanied by enhanced nuclear signal, consistent with the activation of TGF-β-dependent transcriptional responses. In contrast, TNF-α treatment alone did not significantly alter overall KLF11 levels relative to control (mean: 19.94 vs. 18.97; mean difference = −0.9694; 95% CI: −2.820 to 0.8811; adjusted *p* = 0.4633; n = 100), indicating that inflammatory stimulation alone is insufficient to induce KLF11 expression in pHCASMCs. Combined stimulation with TGF-β1 and TNF-α resulted in the highest KLF11 fluorescence intensity (mean: 33.09 vs. 18.97 in control; mean difference = −14.12; 95% CI: −15.97 to −12.27; adjusted *p* < 0.0001; n = 100), significantly exceeding both control and TGF-β1-alone conditions. Prominent nuclear localization was observed in a substantial proportion of cells, suggesting enhanced transcriptional activation under combined cytokine exposure. Collectively, these data demonstrate that KLF11 expression in pHCASMCs is strongly induced by TGF-β1 and markedly amplified under combined TGF-β1 and TNF-α stimulation, whereas TNF-α alone does not significantly regulate KLF11 levels (Figure 8C).

### 2.9. Effects of Cytokine Stimulation on Migration of pHCASMCs in the Wound Healing Assay

**Figure 9:**
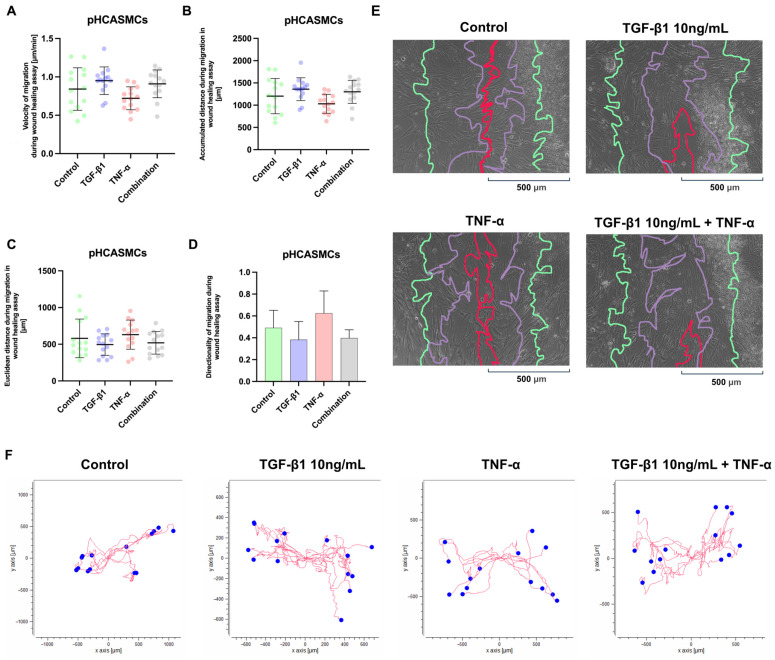
The effects of TGF-β1 and TNF-α on the migration of pHCASMCs in the wound healing assay. The primary human coronary artery smooth muscle cells (pHCASMCs) were stimulated with TGF-β1 (10 ng/mL), TNF-α, or their combination, and cell migration was analyzed. (**A**) Migration velocity and (**B**) accumulated migration distance were significantly higher in the TGF-β1-treated cells compared with the TNF-α-treated cells. (**C**) Euclidean distance did not differ significantly among the experimental groups, indicating comparable net displacement. (**D**) Directionality was significantly increased in the TNF-α-treated cells compared with the TGF-β1 and combination groups, whereas TGF-β1 stimulation was associated with lower directional persistence. Data are presented as mean ± SD; statistical significance was determined using one-way ANOVA followed by Tukey’s multiple comparisons test. The upper panels (**E**) show wound edge contours at successive time points, 3 h (green), 6 h (purple), and 12 h (red), illustrating the dynamics of gap closure. (**F**) The lower panels present individual cell tracking plots; the red lines indicate migration trajectories, and the blue dots mark starting positions. The axes are expressed in micrometers (µm). TGF-β1 stimulation is associated with increased migratory dispersion, whereas TNF-α promotes more directionally oriented movement. Combined treatment results in an intermediate migration pattern.

The migration of the pHCASMCs was evaluated using a wound healing assay, and migration velocity (Figure 9A), accumulated migration distance (Figure 9B), Euclidean distance (Figure 9C), and directionality (Figure 9D) were quantified. The analysis of migration velocity (Figure 9A) revealed a significant difference between experimental groups. TGF-β1 stimulation significantly increased migration velocity compared with TNF-α-treated cells (Tukey’s test: mean diff. = 328.7; 95% CI: 38.00 to 619.4; adjusted *p* = 0.0208). No significant differences were observed between control and TGF-β1 (adjusted *p* = 0.4870), control and TNF-α (*p* = 0.4036), control and combination (*p* = 0.8068), TGF-β1 and combination (*p* = 0.9505), or TNF-α and combination (*p* = 0.0771). These data indicate that TNF-α reduces migration velocity relative to TGF-β1, whereas combined stimulation results in intermediate values comparable to control conditions. Similarly, accumulated migration distance (Figure 9B) was significantly greater in the TGF-β1-treated pHCASMCs compared with the TNF-α-treated cells (mean diff. = −0.2426; 95% CI: −0.4014 to −0.08383; adjusted *p* = 0.0009). In addition, the TNF-α-treated cells exhibited significantly lower accumulated distance compared with the combination group (mean diff. = 0.2275; 95% CI: 0.06872 to 0.3863; adjusted *p* = 0.0021). All remaining pairwise comparisons were not significant (control vs. TGF-β1, *p* = 0.2779; control vs. TNF-α, *p* = 0.1260; control vs. combination, *p* = 0.4086; TGF-β1 vs. combination, *p* = 0.9943). These findings indicate that TNF-α reduces total migratory activity relative to TGF-β1, whereas combined treatment partially restores migration compared with TNF-α alone. In contrast, Euclidean distance (Figure 9C), reflecting net displacement from the point of origin, did not differ significantly among experimental groups (all comparisons ns), indicating that overall displacement remained comparable despite changes in total migratory activity. An assessment of directionality (Figure 9D) demonstrated significant differences between the groups. The TNF-α-treated pHCASMCs exhibited significantly higher directionality compared with the TGF-β1-treated cells (mean diff. = −0.2426; 95% CI: −0.4014 to −0.08383; adjusted *p* = 0.0009). Moreover, directionality in the TNF-α-treated cells was significantly greater than in the combined stimulation group (mean difference = 0.2275; 95% CI: 0.06872 to 0.3863; adjusted *p* = 0.0021). All comparisons were not statistically significant. TGF-β1 enhances the overall migratory activity of pHCASMCs, while TNF-α shifts migration toward greater directional persistence, with combined stimulation producing intermediate wound closure dynamics.

### 2.10. Migration of pHCASMCs Under Confluent Conditions

**Figure 10:**
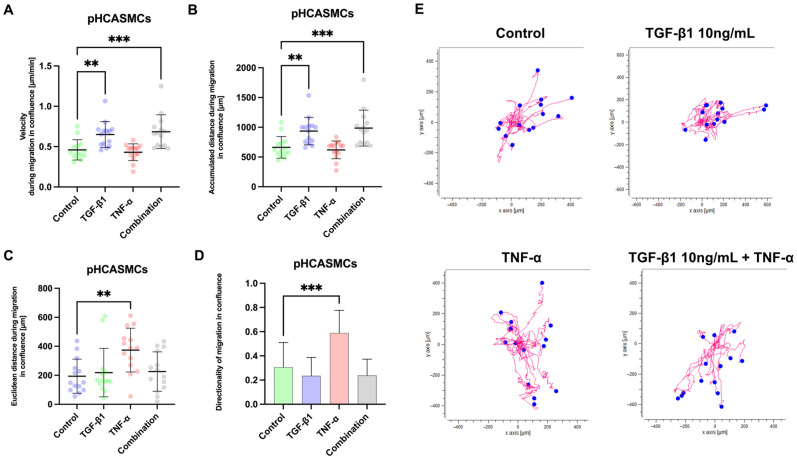
A quantitative analysis of pHCASMC migration under confluent conditions following cytokine stimulation. (**A**) Migration velocity, (**B**) accumulated migration distance, (**C**) Euclidean distance, and (**D**) directionality were quantified in the control cells and after 24 h treatment with TGF-β1 (10 ng/mL), TNF-α (100 ng/mL), or TGF-β1 + TNF-α. TGF-β1 and combined stimulation significantly increased the migration velocity and accumulated distance compared with control (** *p* < 0.01), *** *p* < 0.001), whereas TNF-α alone had no significant effect. The Euclidean distance was significantly reduced only under combined stimulation. The directionality was markedly decreased in the combined treatment group.The data are presented as mean ± SD; statistical analysis was performed using one-way ANOVA followed by Dunnett’s multiple comparisons test. (**E**) Cell tracking plots showing the individual migration paths of the primary human coronary artery smooth muscle cells (pHCASMCs) cultured as a confluent monolayer and treated for 24 h with TGF-β1 (10 ng/mL), TNF-α (100 ng/mL), or TGF-β1 + TNF-α. Each red line represents the trajectory of a single tracked cell, with the blue dots indicating final cell positions relative to the common origin (0, 0). TGF-β1 stimulation increased overall migratory activity, whereas combined cytokine treatment resulted in more dispersed and less directionally persistent movement patterns compared with control. The axes represent displacement in µm.

To evaluate smooth muscle cell motility in a confluent monolayer, migration velocity, accumulated distance, Euclidean distance, and directionality were quantified in pHCASMCs treated with TGF-β1, TNF-α, or their combination. Migration velocity was significantly increased in TGF-β1-treated cells compared with control (mean difference = −0.1903, 95% CI −0.3323 to −0.04839, ** *p* = 0.0058) and was similarly elevated under combined TGF-β1 + TNF-α stimulation (mean difference = −0.2248, 95% CI −0.3667 to −0.08285, *** *p* = 0.0010). TNF-α alone did not significantly affect migration velocity (*p* = 0.9242). Accumulated migration distance showed the same pattern, being significantly increased by TGF-β1 (mean difference = −274.1 µm, 95% CI −478.5 to −69.68, ** *p* = 0.0058) and by combined stimulation (mean difference = −323.7 µm, 95% CI −528.1 to −119.3, *** *p* = 0.0010), whereas TNF-α alone had no significant effect (*p* = 0.9242). Euclidean distance was not significantly altered by TGF-β1 (*p* = 0.9414) or combined treatment (*p* = 0.8887). In contrast, TNF-α significantly increased Euclidean displacement compared with control (mean difference = −180.1 µm, 95% CI −312.0 to −48.25, ** *p* = 0.0049). Similarly, directionality was unchanged in the TGF-β1 (*p* = 0.5521) and combination groups (*p* = 0.5905), whereas TNF-α significantly increased directional persistence relative to control (mean difference = −0.2826, 95% CI −0.4396 to −0.1256, *** *p* = 0.0002). Collectively, under confluent conditions TGF-β1 enhances overall migratory activity (velocity and accumulated distance) without affecting directional parameters, while TNF-α does not increase total motility but promotes greater net displacement and directional persistence. Combined stimulation augments overall migration dynamics without significantly modifying directional behavior relative to control (Figure 10).

### 2.11. Effects of TGF-β1 and TNF-α on Endothelial Tube Formation

**Figure 11:**
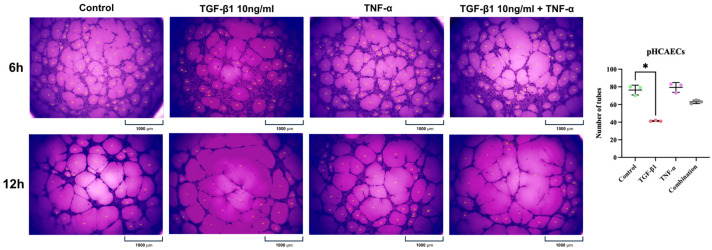
Images of tube formation in pHCAECs following cytokine stimulation (magnification ×40). Representative phase-contrast images of pHCAECs cultured on Matrigel under control conditions or stimulated with TGF-β1 (10 ng/mL), TNF-α (100 ng/mL), or their combination. Images were acquired at 6 h and 12 h after stimulation. At 6 h, control pHCAECs formed an organized and interconnected tubular network. TGF-β1 stimulation was associated with visibly reduced network complexity and decreased branching. TNF-α-treated pHCAECs exhibited a network architecture comparable to control conditions. Combined stimulation resulted in an intermediate phenotype, with partial reduction in tube density compared with control. At 12 h, tube structures appeared thicker and more consolidated across conditions. TGF-β1-treated pHCAECs continued to display reduced network complexity compared with control (* *p* < 0.05), whereas TNF-α maintained a network structure similar to control. Combined treatment resulted in partially preserved network formation relative to TGF-β1 alone. Yellow markers indicate individual tube structures included in quantitative analysis.

A morphological assessment of tube formation revealed clear differences between the experimental conditions. Under control conditions, the endothelial cells formed an extensive and well-organized tubular network. Stimulation with TGF-β1 was associated with visibly reduced network complexity, characterized by fewer and less interconnected tube-like structures. In contrast, the TNF-α-treated cells formed networks comparable to control, with preserved branching and tube density. Combined stimulation with TGF-β1 and TNF-α resulted in an intermediate phenotype, with partial reduction in network complexity compared with control, but less pronounced than that observed with TGF-β1 alone. Quantitative analysis was performed for the 6 h time point. Repeated measures one-way ANOVA with Geisser–Greenhouse correction demonstrated a statistically significant overall treatment effect at 6 h (F(1.088, 2.176) = 96.21, *p* = 0.0077; R^2^ = 0.9796). The mean number of tubes at 6 h was 76.33 under control conditions. TGF-β1 stimulation significantly reduced tube formation to a mean of 41.33 tubes. TNF-α treatment resulted in 79.33 tubes, comparable to the control, whereas combined stimulation yielded 63.00 tubes. Post hoc analysis using Dunnett’s multiple comparisons test showed a significant reduction in tube number in the TGF-β1-treated cells compared with control (mean difference = 35.00; 95% CI: 13.70–56.30; adjusted *p* = 0.0191). No statistically significant differences were observed between control and TNF-α (adjusted *p* = 0.0670) or between control and combined treatment (adjusted *p* = 0.0560). The matching effect (biological replicate variability) was not statistically significant (F(2,6) = 3.964, *p* = 0.0799), indicating that the observed differences were primarily attributable to treatment (Figure 11).

## 3. Discussion

In this study, we investigated how TGF-β1 and TNF-α modulate the migratory potential and functional behavior of pHCAECs and pHCASMCs. These primary-human-coronary-artery-derived cells represent a biologically relevant and translationally robust model, as they preserve key phenotypic and functional characteristics of the human coronary vascular wall and retain physiological responsiveness to inflammatory and remodeling stimuli. Given the central roles of TGF-β1 and TNF-α in vascular inflammation and remodeling, we examined how their individual and combined stimulation affects cell motility across distinct experimental contexts. Particular attention was devoted to KLF11, a transcription factor previously implicated in TGF-β-dependent signaling and vascular homeostasis. Our findings identify KLF11 as a candidate regulator associated with cytokine-induced changes in cellular behavior. However, since direct causal involvement of KLF11 in the observed phenotypic responses was not experimentally demonstrated in this study, its proposed role as an integrator of TGF-β and inflammatory signaling should be considered a hypothesis-generating observation that warrants further mechanistic investigation. Our quantitative analysis of migratory parameters, including trajectory dispersion, velocity, and directional persistence, provides functional insight into the pleiotropic nature of TGF-β1 responses, demonstrating that their outcome depends not only on pathway activation but also on cellular context and structural organization. While our results are consistent with the modulation of signaling pathways commonly associated with TGF-β activity, including SMAD-dependent mechanisms, the present data do not directly establish the specific molecular intermediates responsible for the observed effects. Overall, our findings indicate that cytokine-driven responses are strongly cell-type-specific and depend on the structural and functional state of the cellular monolayer, highlighting differential regulation of endothelial repair mechanisms and smooth muscle remodeling processes. Further studies employing targeted genetic or pharmacological approaches will be required to define the precise signaling mechanisms underlying these responses [12,13].

As previously described, in non-malignant cells canonical TGF-β signaling is tightly constrained by multilayered feedback mechanisms operating at the level of ligand activation, receptor turnover, Smad activity, and Smad-driven transcription, thereby fine-tuning signal amplitude and duration rather than allowing unrestricted pathway escalation [14]. In settings of sustained signaling pressure—most prominently in cancer—this balance can be destabilized, giving rise to the context-dependent functional switch known as the “TGF-β paradox” [15]. In the cardiovascular context, however, negative-feedback control is thought to remain largely preserved in primary coronary vascular cells, supporting a dose-dependent and self-limiting pattern of SMAD2/3 activation. As highlighted by Deng et al. (2024), SMAD activity is dynamically regulated by inhibitory SMADs, ubiquitin ligases, phosphatases, and competing transcriptional cofactors, all of which can reshape signal amplitude and duration independently of ligand abundance [16]. Consistent with this model, increasing the TGF-β1 concentration from 10 to 100 ng/mL was associated with a relative reduction in the nuclear pSMAD2/3 signal, which may reflect engagement of regulatory processes such as feedback modulation or pathway desensitization, rather than a simple monotonic increase in canonical output. This dose–response behavior provided a practical rationale for selecting 10 ng/mL TGF-β1 for subsequent experiments aimed at modeling sustained inflammation-associated TGF-β activity without overwhelming endogenous regulatory mechanisms. Importantly, our data indicate that activation of the canonical TGF-β/SMAD2/3 pathway was observed not only following exogenous TGF-β1 stimulation. TNF-α alone increased nuclear pSMAD2/3 levels in the pHCAECs, and combined treatment further enhanced this effect, suggesting potential functional interaction between inflammatory and canonical TGF-β signaling. Similar observations have been reported in other cellular systems. Li et al. demonstrated that TNF-α treatment significantly upregulated TGF-β1, p-Smad2, and p-Smad3 expression, supporting the possibility of activation of the TGF-β/Smad2/3 axis downstream of inflammatory stimulation [17]. Likewise, Yoshimatsu et al. showed that co-stimulation with TGF-β and TNF-α resulted in sustained Smad2/3 activation accompanied by the increased expression of TGF-β family signaling components. Together, these findings are consistent with the notion that TNF-α can modulate canonical Smad2/3 activation under inflammatory conditions [18]. Chen et al. described cell-type-specific TGF-β signaling programs primarily at the transcriptomic level in vivo, whereas our study focuses on proximal canonical pathway activity assessed at the protein level by nuclear pSMAD2/3 accumulation in primary human coronary endothelial and smooth muscle cells. Because SMAD phosphorylation and nuclear retention are influenced by multiple regulatory inputs, pSMAD2/3 intensity should be interpreted cautiously as an indicator of pathway engagement rather than a direct measure of transcriptional output or functional phenotype. In line with this concept, we observed that TNF-α-induced canonical activation in pHCAECs did not uniformly correspond to identical biological outcomes across cell types or assay contexts. In endothelial cells, TNF-α predominantly promoted inflammatory activation, whereas TGF-β1 primarily affected cytoskeletal organization and migration dynamics, and combined stimulation produced the most coordinated wound closure. In smooth muscle cells, TGF-β1 enhanced overall motility, while TNF-α preferentially influenced directional parameters; importantly, combined stimulation markedly amplified KLF11 expression beyond TGF-β1 alone. These observations suggest that inflammatory cues may reshape canonical SMAD2/3 signaling responses and highlight that pathway activation does not necessarily translate into a single predictable functional outcome [19].

Our findings further support the view that canonical TGF-β/SMAD2/3 signaling in vascular smooth muscle cells does not uniformly produce a pro-remodeling phenotype. Although TGF-β1 increased migration velocity and accumulated distance in confluent pHCASMCs, it did not improve directional efficiency, indicating enhanced activity without coordinated displacement. TNF-α alone reduced Euclidean distance and directionality, consistent with less organized movement despite detectable pathway activation. Combined stimulation increased overall migratory dynamics while maintaining displacement parameters at control levels. Together, these results suggest that in primary human coronary smooth muscle cells the functional consequences associated with canonical pathway activation depend on the inflammatory context and migration mode, rather than reflecting a simple linear relationship between pathway activation and remodeling-related behavior [20].

In the present study, KLF11 was identified as a context-dependent transcription-associated factor showing differential nuclear localization patterns in response to inflammatory and remodeling-related stimuli in coronary endothelium and smooth muscle. In endothelial cells, KLF11 induction—particularly its pronounced nuclear enrichment under combined stimulation—was observed in association with coordinated migratory adaptation rather than uncontrolled inflammatory escalation, consistent with a potential contribution to maintaining functional plasticity under cytokine stress. In smooth muscle cells, KLF11 nuclear signal intensity was predominantly responsive to TGF-β stimulation and was further enhanced by inflammatory co-stimulation, consistent with context-dependent changes in localization associated with remodeling-related cellular responses. These observations are compatible with the hypothesis that KLF11 may participate in context-dependent transcriptional responses to combined inflammatory and profibrotic cues, although the present study relied on quantitative immunofluorescence measurements and was not designed to establish a direct causal or mechanistic role of KLF11 in the observed phenotypic changes. Accordingly, the observed changes in the KLF11 signal are interpreted as reflecting relative differences in nuclear localization patterns rather than direct quantification of protein abundance. This interpretation is consistent with previous studies describing KLF11 as a vasculoprotective factor in specific experimental models. Liang et al. demonstrated that VSMC-specific Klf11 deletion accelerates arterial thrombosis through the transcriptional upregulation of tissue factor (F3), whereas KLF11 directly represses the F3 promoter [21]. In intracranial aneurysm models, KLF11 overexpression improves VSMC viability and reduces inflammatory responses under oxidative stress conditions [22]. Together with reports that KLF11 attenuates cardiac hypertrophy and fibrosis, these findings support the broader view that KLF11 may function as a context-dependent regulatory factor within the cardiovascular system [23]. From a functional perspective, this interpretation is consistent with the established characterization of KLF11 as a TGF-β-inducible transcription factor implicated in transcriptional regulation, including through interactions with chromatin-modifying complexes [24].

Notably, the effects of TGF-β1 on endothelial tube formation observed in our study differ from those reported in proliferative endothelial models. In primary human coronary endothelial cells, TGF-β1 reduced network complexity despite promoting cytoskeletal remodeling and increased migratory activity, consistent with features of structural destabilization rather than enhanced morphogenetic capacity. This discrepancy may reflect differences in cellular context between proliferative and mature endothelial phenotypes [25]. Structural observations support this interpretation, as TGF-β1 reduced VE-cadherin continuity at intercellular junctions, an effect further intensified by combined cytokine stimulation. These findings are consistent with dynamic junctional remodeling rather than sustained endothelial cohesion [26]. Finally, the VCAM-1 expression analysis further underscores the context-dependent nature of TGF-β signaling in coronary endothelial cells. TNF-α robustly induced VCAM-1 expression, whereas TGF-β1 alone had minimal effect, and combined stimulation maintained high expression levels. These results suggest that TGF-β1 did not measurably attenuate TNF-α-associated endothelial activation under the inflammatory conditions examined in this study [27]. Notably, TNF-α-dependent VCAM-1 regulation varies across vascular cell types and physiological contexts, which may contribute to the variability reported in other experimental systems [27,28].

## 4. Materials and Methods

### 4.1. Cell Culture and Treatment

Primary human coronary artery endothelial cells (pHCAECs) and primary human coronary artery smooth muscle cells (pHCASMCs) were obtained from healthy donors. These primary coronary artery cell types were chosen to ensure physiological relevance and to more accurately model cellular responses characteristic of the human coronary vasculature. The cells were purchased from the American Type Culture Collection (ATCC, Manassas, VA, USA) and cultured in accordance with the manufacturer’s recommendations. The endothelial cells were maintained in vascular cell basal medium (ATCC) supplemented with an endothelial cell growth kit containing recombinant human VEGF (5 ng/mL), EGF (5 ng/mL), basic FGF (5 ng/mL), IGF-1 (15 ng/mL), L-glutamine (10 mM), heparin sulfate (0.75 U/mL), hydrocortisone hemisuccinate (1 µg/mL), fetal bovine serum (2%), ascorbic acid (50 µg/mL), and antibiotics (penicillin 10 U/mL, streptomycin 10 µg/mL, and amphotericin B 25 µg/mL). The smooth muscle cells were cultured in vascular cell basal medium supplemented with a vascular smooth muscle cell growth kit containing recombinant human basic FGF (5 ng/mL), insulin (5 µg/mL), EGF (5 ng/mL), L-glutamine (10 mM), ascorbic acid (50 µg/mL), fetal bovine serum (5%), and the same antibiotic mixture. The cells were seeded at a density of 5000 viable cells/cm^2^ in T-25 culture flasks and maintained at 37 °C in a humidified atmosphere with 5% CO_2_. After reaching confluence, the cells were treated with complete growth medium supplemented with recombinant human TNF-α (100 ng/mL; Sigma-Aldrich, St. Louis, MO, USA) expressed in HEK293 cells, for 24 h. In parallel experiments, the cells were stimulated with recombinant human TGF-β1 (10 ng/mL; human recombinant protein expressed in HEK293 cells) for 24 h. The control cells were maintained under identical conditions without cytokine treatment. Only cells between passages three and four were used for all experiments. Bright-field images of cultured cells were captured using an Olympus CKX53 inverted microscope (Olympus, Tokyo, Japan) equipped with a ×20 air objective and an Olympus EP50 digital camera. Image acquisition was performed using the EPview software version V3.7.7_20230531 (Olympus, Tokyo, Japan).

### 4.2. Cell Viability Assay

The cells were cultured under standard conditions in accordance with the manufacturer’s recommendations and maintained in complete growth medium at 37 °C in a humidified atmosphere with 5% CO_2_. The cells were seeded into 24-well plates at an appropriate density to reach near-confluence at the time of treatment and allowed to attach overnight. After confluence was reached, the cells were cultured for an additional period of 24 h in complete vascular growth medium supplemented or not with 100 ng/mL rh TNFα and 10 ng/mL rh TGF-β. Following experimental stimulation, cell metabolic activity was assessed using the MTT (3-(4,5-dimethylthiazol-2-yl)-2,5-diphenyltetrazolium bromide) colorimetric assay. A stock solution of MTT was prepared by dissolving MTT powder in sterile PBS to a final concentration of 5 mg/mL, followed by incubation at 37 °C with intermittent mixing until fully dissolved. Immediately before use, the MTT stock solution was diluted 1:10 in complete culture medium to obtain the working solution. The culture medium was removed, and the cells were incubated with 1 mL/well of MTT working solution for 2 h at 37 °C. After incubation, the MTT solution was carefully aspirated and replaced with an equal volume of dimethyl sulfoxide (DMSO) (1 mL/well) to dissolve the formazan crystals. The plates were gently pipetted to ensure complete solubilization. Absorbance was measured using a microplate spectrophotometer at 570 nm. A blank sample containing DMSO alone (without cells) was included, and its absorbance was subtracted from all experimental readings. The results were expressed as relative absorbance values normalized to control cells, which were set to 100%.

### 4.3. Immunofluorescence Staining and Localization of Proteins

For fluorescence localization of the selected proteins, the primary human coronary artery endothelial cells (pHCAECs) were seeded onto sterile 18 mm glass coverslips (Thermo Fisher Scientific, Waltham, MA, USA) placed in 12-well plates (Corning, Corning, NY, USA) and cultured under standard conditions (37 °C and 5% CO_2_) until reaching confluence, followed by 24 h incubation in complete vascular growth medium under control conditions or stimulation with TGF-β1 (10 ng/mL), TNF-α (100 ng/mL), or their combination; the cells were fixed with 4% formaldehyde for 15 min at room temperature, washed with PBS, permeabilized with 0.25% Triton X-100 for 10 min, and blocked with 3% BSA for 45 min, then incubated for 1 h at room temperature with primary antibodies including rabbit anti-VE-cadherin polyclonal antibody (PA5-19612, Thermo Fisher Scientific; 1:200), rabbit anti-phospho-SMAD2 (Ser465/467)/SMAD3 (Ser423/425) polyclonal antibody (PA5-110155, Invitrogen/Thermo Fisher Scientific, Waltham, MA, USA; 1:100) detecting activated SMAD2/3, rabbit anti-FKLF/KLF11 polyclonal antibody (BS-16096R, Bioss, Beijing, China; 1:200), and mouse recombinant monoclonal anti-VCAM-1 (clone SA05-04, MA5-31965, Thermo Fisher Scientific; 1:200), followed by incubation with appropriate Alexa Fluor-conjugated secondary antibodies (1:200, Thermo Fisher Scientific) for 1 h at room temperature, staining of F-actin with phalloidin–Alexa Fluor 488 (1:40, 20 min), nuclear counterstaining with DAPI for 10 min, mounting in Aqua-Poly/Mount (Polysciences, Warrington, PA, USA), and imaging using a Nikon C1 laser scanning confocal microscope (Nikon, Tokyo, Japan) equipped with a Plan VC Apo ×100 oil objective and EZ-C1 3.80 software, with excitation at 408 nm (DAPI), 488 nm (Alexa Fluor 488), and 543 nm (Alexa Fluor 594), maintaining identical acquisition settings within each experimental set, and fluorescence intensity quantified in ImageJ software version 1.54g (National Institutes of Health, Bethesda, MD, USA) within manually defined nuclear or membrane regions of interest with background subtraction.

### 4.4. Wound Healing Migration

Cell migration was assessed using a wound healing assay performed with two-well culture inserts (Ibidi, Gräfelfing, Germany) placed in six-well plates (Corning). The primary human coronary artery endothelial cells (pHCAECs) were resuspended in 70 μL of complete vascular growth medium and seeded at a density of 1100 cells per reservoir into each insert. The cells were cultured under standard conditions (37 °C and 5% CO_2_) until a fully confluent monolayer was formed. After reaching confluence, the inserts were carefully removed using sterile forceps to create a defined cell-free gap. The wells were gently rinsed with DPBS to eliminate detached cells and debris. The cells were then maintained in complete vascular growth medium under the following experimental conditions: control (no cytokine treatment), TGF-β1 (10 ng/mL), TNF-α (100 ng/mL), or combined TGF-β1 (10 ng/mL) + TNF-α (100 ng/mL). Time-lapse phase-contrast images were acquired at 10 min intervals for 24 h using an Axio Observer Z1 inverted motorized microscope (Zeiss) equipped with a live-cell incubation system (PeCon), an EC Plan-Neofluar ×10/0.30 Ph1 air objective, an Axiocam 503 mono camera, and ZEN 2 software (Zeiss, Oberkochen, Germany). The images were recorded at the region of the migration gap under standard culture conditions. Individual cell trajectories were manually tracked using the Manual Tracking plugin in ImageJ (NIH). A quantitative analysis of migration parameters, including migration velocity, accumulated distance, Euclidean distance, and directionality, was performed using the Chemotaxis and Migration Tool 2.0 (Ibidi, Gräfelfing, Germany).

### 4.5. Confluent Cell Migration

Confluent cell migration was assessed in six-well plates (Corning). Primary human coronary artery endothelial cells (pHCAECs) were cultured under standard conditions until reaching full confluence. Following confluence, cells were maintained in complete vascular growth medium under the following experimental conditions: control (no cytokine treatment), TGF-β1 (10 ng/mL), TNF-α (100 ng/mL), or combined TGF-β1 (10 ng/mL) + TNF-α (100 ng/mL). Time-lapse phase-contrast images were acquired at 10 min intervals over 36 h under standard culture conditions using an Axio Observer Z1 inverted motorized microscope (Zeiss) equipped with a live-cell incubation system (PeCon), an EC Plan-Neofluar ×10/0.30 Ph1 air objective, an Axiocam 503 mono camera, and ZEN 2 software (Zeiss). The images were recorded in selected regions of the confluent monolayer to monitor spontaneous cell movement within a dense cellular layer. Individual cell trajectories were manually tracked using the Manual Tracking plugin in ImageJ (NIH). Migration parameters, including migration velocity, accumulated distance, Euclidean distance, and directionality, were calculated from tracked trajectories using the Chemotaxis and Migration Tool 2.0 (Ibidi, Gräfelfing, Germany).

### 4.6. In Vitro Tube Formation Assay

Endothelial tube formation was assessed using μ-Slide Angiogenesis chambers (Ibidi) in accordance with the manufacturer’s instructions. Briefly, each inner well was coated with 10 μL of ice-cold, growth-factor-reduced, phenol-red-free Matrigel Basement Membrane Matrix (Corning) and allowed to polymerize for 30 min at 37 °C. Primary human coronary artery endothelial cells (pHCAECs) were resuspended in complete vascular growth medium and seeded at a density of 10,000 cells per well in a final volume of 50 μL. The cells were cultured under the following experimental conditions: control (no cytokine treatment), TGF-β1 (10 ng/mL), TNF-α (100 ng/mL), or combined TGF-β1 (10 ng/mL) + TNF-α (100 ng/mL). The slides were maintained under standard culture conditions (37 °C, 5% CO_2_). Tube formation was monitored by time-lapse phase-contrast imaging at 10 min intervals over 24 h using an Axio Observer Z1 inverted motorized microscope (Zeiss) equipped with a live-cell incubation system (PeCon), an EC Plan-Neofluar ×10/0.30 Ph1 air objective, an Axiocam 503 mono camera, and ZEN 2 software (Zeiss, Oberkochen, Germany). The images were recorded at the same predefined location for each well to ensure consistency of analysis. Additionally, bright-field images were acquired at defined time points (3, 6, 12, and 24 h) to evaluate network progression and structural stability. For end-point documentation, the cells were fixed with 4% formaldehyde for 15 min at room temperature, washed three times with PBS, and stained with crystal violet. Bright-field images were captured using an Olympus CKX53 inverted microscope (Olympus) equipped with a ×4 air objective and an Olympus EP50 digital camera. Image acquisition was performed using the EPview software version V3.7.7_20230531 (Olympus, Tokyo, Japan). Quantitative analysis was carried out by manually counting the number of tube-like structures using ImageJ software version 1.54g (National Institutes of Health, Bethesda, MD, USA).

### 4.7. Statistical Analysis

Statistical analyses were performed using GraphPad Prism software (GraphPad 8 Software, San Diego, CA, USA). Data are presented as mean ± standard deviation (SD). Comparisons among multiple groups were performed using ordinary one-way ANOVA followed by Tukey’s or Dunnett’s multiple comparisons test, as appropriate. For time-dependent tube formation experiments, repeated measures one-way ANOVA with Geisser–Greenhouse correction was applied. For migration analyses (velocity, accumulated distance, Euclidean distance, and directionality), statistical comparisons between experimental groups were performed using one-way ANOVA with appropriate post hoc testing. Cell trajectories were analyzed using the Chemotaxis and Migration Tool 2.0 (Ibidi). The Rayleigh test was used to assess the homogeneity of directional cell movement where applicable. A *p*-value < 0.05 was considered statistically significant and indicated in figures as * *p* < 0.05; ** *p* < 0.01; *** *p* < 0.001; **** *p* < 0.0001.

## 5. Conclusions

TGF-β1 and TNF-α differentially regulate the migration and functional behavior of primary human coronary endothelial and smooth muscle cells in a strongly cell-type- and context-dependent manner. TGF-β1 was associated with enhanced cytoskeletal remodeling and increased overall motility, whereas TNF-α was linked to changes in directional persistence and cellular polarization. In endothelial cells, TGF-β1 was associated with increased migratory activity but reduced tube formation, consistent with features of a shift toward a more contractile–mesenchymal phenotype, which appeared to require inflammatory co-stimulation to achieve coordinated wound closure. In smooth muscle cells, TGF-β1 was associated with migration patterns characteristic of remodeling-related responses, while TNF-α modulated directional displacement without inducing strong inflammatory activation under the conditions examined. KLF11 was identified as a context-sensitive transcription-associated factor showing differential nuclear localization in response to combined cytokine stimulation, suggesting a potential role in adaptive stress responses in endothelium and TGF-β-related remodeling processes in smooth muscle. However, the precise functional role of KLF11 in these processes remains to be established and warrants further investigation using dedicated functional and mechanistic approaches. Collectively, our findings indicate that canonical SMAD2/3 activation alone is not sufficient to reliably predict functional outcomes, underscoring the pleiotropic and context-dependent nature of TGF-β signaling in the coronary artery wall.

## Data Availability

The original contributions presented in the study are included in the article; further inquiries can be directed to the corresponding author.
